# Interleukin-29 modulates proinflammatory cytokine production in synovial inflammation of rheumatoid arthritis

**DOI:** 10.1186/ar4067

**Published:** 2012-10-19

**Authors:** Fang Wang, Lingxiao Xu, Xiaoke Feng, Dunming Guo, Wenfeng Tan, Miaojia Zhang

**Affiliations:** 1 Department of Cardiology, the First Affiliated Hospital of Nanjing Medical University, 300 Guangzhou Road, Nanjing 210029, China; 2 Department of Rheumatology, the First Affiliated Hospital of Nanjing Medical University, 300 Guangzhou Road, Nanjing 210029, China; 3 Department of Orthopaedics, the First Affiliated Hospital of Nanjing Medical University, 300 Guangzhou Road, Nanjing, 210029, China

## Abstract

**Introduction:**

The immunoregulatory function of interleukin (IL)-29 has recently been recognized. However, little is known about the involvement of IL-29 in the pathogenesis of rheumatoid arthritis (RA). This study aimed to examine the expression profiles of IL-29 in blood, synovial fluid (SF) and synovium in RA patients and investigate the effect of IL-29 on cytokines production in RA synovial fibroblasts.

**Methods:**

The transcript levels of IL-29 and its specific receptor IL-28Rα in peripheral blood mononuclear cells (PBMC) and synovium were determined by real-time reverse transcription-polymerase chain reaction (real-time PCR). The concentrations of IL-29 in serum and synovial fluid (SF) were quantified by enzyme-linked immunoassay (ELISA), and the correlation of serum IL-29 levels with disease activity in RA patients was investigated. Furthermore, the expression of IL-29 in RA synovium was examined by immunohistochemistry and double immunofluorescence analysis. Finally, the expression of IL-6, IL-8, IL-10, IL-17 and matrix metalloproteinase-3 (MMP-3) in synovial fibroblasts upon IL-29 stimulation was determined by real-time PCR.

**Results:**

IL-29 and IL-28Rα mRNA expression in PBMC was significantly increased in patients with RA compared with healthy controls (HC). The serum levels of circulating IL-29 were higher in RA than those in HC. Increased IL-29 levels were detected in RA SF when compared with osteoarthritis (OA) SF. However, serum IL-29 levels showed no significant correlation with RA disease activity. IL-29 was mostly expressed in the lining region of RA synovium. Moreover, IL-29 was expressed predominately in synovial macrophages and fibroblasts. RA synovial fibroblasts exposed to IL-29 specifically upregulated IL-6, -8 and MMP-3 but downregulated IL-10.

**Conclusions:**

The findings in the present study indicate, for the first time, that IL-29 is dysregulated in patients with RA, which may contribute to the RA pathogenesis via inducing the production of proinflammatory cytokines, chemokines or matrix metalloproteinases in synovial fibroblasts.

## Introduction

Rheumatoid arthritis (RA) is characterized by chronic inflammation, articular destruction and abnormal immune response. Although the pathogenesis of RA remains unclear, the accumulated evidence has suggested that cytokines play an important role in the development and maintenance of RA disease activity. In the past decade, numerous studies have shown that a variety of cytokines including TNF-α, IL-1α, -1β, -6, -7, -15, -17, -18, -21, -23, -32, and -33 contribute to RA pathogenesis [1]. Consequently, biologics that target TNF-α or IL-6 for the treatment of RA have been extensively studied and have profoundly changed RA treatment strategy. Considering about 30% of RA patients could experience an inadequate response to current biologics, it is still a challenge to identify key cytokines involved in RA. Recently, the upregulation of interferon-inducible genes has been found in the synovial lining regions and whole blood of patients with RA, suggesting that interferons (IFNs) may also play an important role in the pathogenesis of RA [2,3].

The classical interferon (IFN) family cytokines are known to be critically involved in both innate and adaptive immune responses during viral infection and autoimmune inflammation. The IFN family includes three subfamilies (type I, type II and type III). Type I IFNs include IFN-α, β, ω, κ, ε, τ, ζ, δ and ν subtypes [4], whereas type II IFNs are represented by IFN-γ. Type III IFNs consist of three newly identified members, IL-29 (IFN-λ1), IL-28A (IFN-λ2) and IL-28B (IFN-λ3) [3,5]. Type III IFNs closely resemble the type I IFNs in terms of expression after virus infection as well as intracellular signaling and activation of antiviral host factors in susceptible cells [6]. However, the striking differences between type I and III IFNs include the cell-type and tissue-specific distribution of their respective receptor complexes [7]. Type I IFNs signal through a universally expressed cell surface receptor complex composed of two subunits, IFNAR1 and IFNAR2 [8]. By contrast, type III IFNs act through a cell surface receptor composed of a unique IL-28 receptor α chain (IL-28Rα, also known as IFNLR1) and IL-10R2 chain that is also the subunit of the receptor of IL-10, IL-22 and IL-26 [9]. The specific activity of type III IFNs is determined in part by the expression level of its receptor chain IL-28Rα, which is expressed on a limited range of tissues and cell types, such as lung, heart, liver and prostate tissues, dendritic cells, A549 and HeLa S3 cell lines [7,10,11]. Recent studies have revealed the unique biological activities of type III IFNs in and beyond innate antiviral immunity [12,13]

IL-29 shows the highest level of activity among three members of type III IFNs and has exhibited its potential immunoregulatory function. Recent studies have reported that IL-29 acts an inhibitor of human Th2 responses and modulates the Th1/Th2 response by the diminution of IL-13 secretion *in vitro *[14,15]. It has been shown that IL-29 specifically upregulates the levels of IL-6, IL-8 and IL-10 secreted by monocytes [16] and also enhances IL-2-dependent proliferation of CD4+CD25+Foxp3+ T cells induced by dendritic cells [17]. Interestingly, a recent study has reported that IL-29 plays an important role in the pathogenesis of systemic lupus erythematosus (SLE) by inducing the secretion of chemokines IP-10, MIG and IL-8 in peripheral blood mononuclear cells [18,19]. Although accumulated data have indicated a crucial role of IL-29 in modulating immune response and enhancing inflammatory reaction, whether IL-29 is involved in the pathogenesis of RA remains unclear.

In this study, we investigated whether the expression of IL-29 is dysregulated in patients with RA. We found significantly elevated levels of IL-29 in peripheral blood mononuclear cells (PBMC), serum and synovial fluid (SF) of RA patients compared with healthy controls or osteoarthritis (OA) patients. Moreover, we identified both synovial macrophages and fibroblasts as the major cellular source for IL-29 expression in RA synovial tissue. Furthermore, our *in vitro *studies revealed that recombinant IL-29 can stimulate MH7A cells, a human synovial fibroblast cell line, for enhanced production of proinflammatory cytokines, indicating a novel function of IL-29 in driving synovial inflammation during RA development. Our results further support the hypothesis that IL-29 may play a previously unrecognized role in the pathogenesis of RA.

## Materials and methods

### Reagents

Human IL-29 ELISA reagent kits were purchased from Adlitteram Diagnostic Laboratories (San Diego, CA, USA). Rabbit anti-human IL-29/IL-28Rα polyclonal antibody, and mouse anti-human CD68 monoclonal antibody were purchased from Abcam (Cambridge, MA, USA), mouse anti-human fibroblast growth factor-basic (FGF-2) monoclonal antibody from Millipore (Billerica, MA, USA), recombinant human IL-29 and TNF-α were from Peprotech (Rocky Hill, NJ, USA). Donkey anti-rabbit IgG-R and goat anti-rabbit IgG/TRITC were from Santa Cruz Biotechnology (Santa Cruz, CA, USA). DyLight™488-conjugated AffiniPure donkey anti-mouse IgG, peroxidase-conjugated sheep anti-rabbit secondary antibody and peroxidase-conjugated sheep anti-mouse secondary antibody were from Jackson Immunoresearch (West Grove, PA, USA). The Diaminobenzidine (DAB) substrate kit was from Dako (Glostrup, Denmark). PrimeScript™ RT Master Mix and SYBR Green PCR Master Mix were obtained from Takara Biotechnology (Dalian, China). Dulbecco's modified Eagle's medium (DMEM) was from Gibco (Carlsbad, CA, USA) and fetal bovine serum (FBS) from Biosource International (Camarillo, CA, USA).

### Patients and samples

Patients with RA, OA and healthy control (HC) patients were recruited randomly from the First Affiliated Hospital of Nanjing Medical University. Blood samples were collected from 54 RA patients and 60 HC. SF samples were obtained from 20 RA and 20 OA patients. Synovium samples were obtained from six RA patients and five HC after therapeutic synovectomy or amputation. The classification of RA fulfilled the American College of Rheumatology (ACR)/European League Against Rheumatism (EULAR) 2009 diagnostic criteria [20]. The general characteristics of both patients and controls subjects are summarized in Table 1. All patients were recruited from the baseline and had not yet been treated with disease-modifying anti-rheumatic drugs (DMARDs) and/or steroids before their blood samples were collected. This study was approved by the Ethical Committee of the First Affiliated Hospital of Nanjing Medical University, and all donors provided written informed consent.

**Table 1:** Clinical and laboratory characteristics in patients with rheumatoid arthritis (RA) and osteoarthritis (OA), and healthy control (HC) subjects.

Parameter	RA (n = 54)	OA (n = 20)	HC (n = 60)
Age (y)	48.2 (17-65)	61 (57-82)	43.4 (24-64)
Sex (female/male)	34/6	24/4	20/5
Median age at onset (y)	40 (13-63)	-	-
Median disease duration (m)	36 (3-444)	-	-
Number of tender joints	6.50 (0-28)	-	-
Number of swollen joints	1.50 (0-28)	-	-
ESR (mm/h)	37.5 (10-125)	-	-
CRP (mg/l)	10.32 (2.97-84.5)	-	-
RF (U/I)	76.4 (10.3-954)	-	-
DAS28-ESR	4.58 ± 2.08	-	-
BMI (kg/m^2^)	22.45 (16.65-28.55)	-	22.91 (17.33-26.17)
Total cholesterol (mmol/l)	4.41 (2.55-6.16)	-	5.19 (3.9-7.1)
Triglycerides (mmol/l)	1.44 (0.49-2.15)	-	1.46 (0.84-3.11)

The values shown represent the median and the ranges. BMI, body mass index; CRP C-reactive protein; DAS28, 28-joint Disease Activity Score; ESR, erythrocyte sedimentation rate; RF, rheumatoid factor.

Blood samples were collected from peripheral veins. PBMCs were isolated by Ficoll-Hypaque density centrifugation. Serum and SF samples were stored at -80ºC. Synovial biopsies were stored in liquid nitrogen for mRNA analysis or in Carnoy's fixative for histological analysis.

### Real-time PCR

Total RNA was extracted from PBMC, synovial tissue and fibroblasts using TRIzol (Invitrogen, Carlsbad, CA, USA). Reverse transcription reaction was conducted at 37ºC for 15 min, 85ºC for 5 sec in a 20 μL mixture containing 1 μg of total RNA, and PrimeScript™ RT Master Mix. Each real-time PCR was prepared in a 20 μL reaction mixture containing 10 μL SYBR Green PCR Master Mix, 1 μL cDNA, 0.8 μL primers (200 nM each of forward and reverse primers), and conducted on a ABI Prism 7900 sequence detector (Applied Biosystems, Carlsbad, CA, USA). Cycling conditions consisted of initial denaturation 30 sec at 95ºC, followed by 40 cycles of 5 sec at 95ºC, and 30 sec at 60ºC. The primer sequences are summarized in Table 2. All samples of RA and controls were assayed in triplicate. Relative gene expression was determined by the 2^-ΔΔct ^method.

**Table 2:** List of the sequence of gene primers.

Gene name	Forward (5' to 3')	Reverse (5' to 3')
Human IL-29	GAAGCAGTTGCGATTTAGCC	GAAGCTCGCTAGCTCCTGTG
Human IL-28Rα	CCTCCCCAGAATGTGACGC	GGAGCCATGTCAGGTACACG
Human IL-6	AACCTGAACCTTCCAAAGATGG	TCTGGCTTGTTCCTCACTACT
Human IL-8	CATACTCCAAACCTTTCCACCCC	TCAGCCCTCTTCAAAAACTTCTCCA
Human IL-10	CAAATGAAGGATCAGCTGGACAA	GCATCACCTCCTCCAGGTAAAAC
Human IL-17	CCCGGACTGTGATGGTCAAC	GCACTTTGCCTCCCAGATCA
Human MMP-3	CAGGCTTTCCCAAGCAAATA	TTGCATTTGGGTCAAACTCC
Human GAPDH	AGAAGGCTGGGGCTCATTTG	AGGGGCCATCCACAGTCTTC

### ELISA

Levels of IL-29 in serum and SF were measured by ELISA according to the manufacturer's instructions. The correlation was analyzed between IL-29 and laboratory values, including erythrocyte sedimentation rate (ESR), C-reactive protein (CRP), anti-cyclic citrullinated peptides (CCP) and rheumatoid factor (RF) in serum of RA patients.

### Immunohistochemical analysis

Specimens were fixed in Carnoy's fixative and embedded in paraffin wax. Paraffinized synovial tissues were sectioned to 3μm thickness, deparaffinized in xylene and rehydrated through a series of concentrations of ethanol. After inactivation of endogenous peroxidase, sections were blocked by incubation with 5% bovine serum album for 30 min at room temperature, then incubated with rabbit anti-human IL-29 antibody at 4ºC overnight in a humidified chamber. After washing, sections were next incubated with peroxidase-conjugated goat anti-rabbit secondary antibody for 1 h at room temperature. The reactions were developed using a DAB substrate kit, with hematoxylin as counterstain. Each slide was evaluated by one of the authors (Ms. Xiaoke Feng) under a microscope (Nikon, Tokyo, Japan). Tissue sections were scored for staining of the lining on a 0 to 5 scale [21] as follows: 0 = no staining, 1 = few of the cells positively stained, 2 = some (fewer than half) of the cells stained, 3 = approximately half of the cells stained, 4 = more than half of the cells stained, and 5 = all cells stained. For each section, the number of positively stained cells was counted in 20 fields.

For double immunofluorescence labeling, tissue sections were incubated with a mixture of primary antibodies (rabbit anti-IL-29 pAb, mouse anti-CD68 or FGF-2 mAb) at 4 ºC overnight. Slides were next reacted with a mixture of donkey anti-rabbit IgG-R, DyLight™488-conjugated donkey anti-mouse IgG, and 40, 6-diamidine-20-phenylindole dihydrochloride (DAPI) for 1 h. Images were acquired and processed digitally under a fluorescence microscope (Nikon, Tokyo, Japan).

### Cell lines and treatment

MH7A was a generous gift from Dr. David Yu (UCLA, USA), which was isolated from intra-articular soft tissues of the knee joints of RA patients and established by transfection with the SV40 T antigen [22]. Cells were cultured in DMEM supplemented with 10% FBS, penicillin and streptomycin in a humidified atmosphere of 5% CO_2 _at 37ºC.

Cells were exposed to recombinant human IL-29 (rIL-29) at various concentrations (1, 10 and 100 ng/ml) or TNF-α (10 ng/ml). At 24 h and 48 h following incubation, cells were collected for the detection of IL-6, IL-8, IL-10, IL-17 and matrix metalloproteinase-3 (MMP-3) by real-time PCR.

### Immunofluorescence staining of IL-28Rα in cells

The biological activity of IL-29 was determined in part by the expression level of its specific receptor chain IL-28Rα. MH7A cells were washed in PBS twice for 1 min and then fixed with 4% paraformaldehyde for 15 min. The cells were incubated with rabbit anti-human IL-28Rα antibody for 1 h in 37ºC. After incubation, the cells were washed twice and further incubated with goat anti-rabbit IgG/TRITC for 1 h in room temperature. Finally, the cells were washed and incubated with DAPI staining solution for 2 min, and analyzed by fluorescence microscopy (Nikon, Tokyo, Japan). IL-28Ra was stained red and nuclei were stained in blue.

### Statistical analysis

Statistical analyses were performed with SPSS version 18.0 software (SPSS, Chicago IL, USA). Data were expressed as mean ± SD. Differences between two groups were performed with Student's *t *test for parametric data and Mann-Whitney *U *test for nonparametric data. The Pearson correlation test was used to evaluate the correlation between serum IL-29 levels and laboratory values and clinical features. For all experiments, *P *<0.05 was considered as significant.

## Results

### Upregulated expression of IL-29 and its receptor transcripts in PBMCs from RA patients

**Figure 1:**
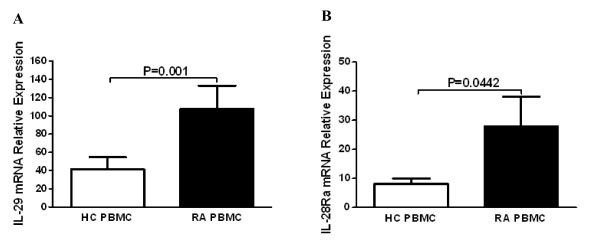
**Increased expression of IL-29 and IL-28Rα in rheumatoid arthritis (RA) peripheral blood mononuclear cells (PBMC) compared to in healthy control (HC) PBMC**. Levels of mRNA for IL-29 **(A) **and IL-28Rα **(B) **in PBMC from RA (n = 40) and HC (n = 40) were determined by real-time PCR. Relative gene expression was determined by the 2^-ΔΔct ^method. Values are the mean ± SD. Actual *P *values are shown in the graph.

To explore whether IL-29 was involved in the pathogenesis of RA, we first examined the expression of IL-29 mRNA and its receptor IL-28Rα in PBMC by real-time PCR. It was found that expression of IL-29 and IL-28Rα mRNA was significantly higher in RA PBMCs when compared to HC (*P *= 0.001 and 0.0442, respectively; Figure 1A, B).

### Increased serum and SF levels of IL-29 in RA patients

**Figure 2:**
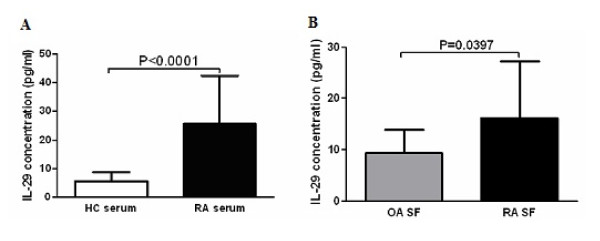
**Increased expression of IL-29 in rheumatoid arthritis (RA) serum and synovial fluid (SF) compared to in healthy control (HC) serum and osteoarthritis (OA) SF**. Levels of IL-29 in serum **(A) **from RA (n = 54) and HC (n = 60), and SF **(B**) from RA (n = 20) and OA (n = 20) were quantified by ELISA. Values are the mean ± SD. Actual *P *values are shown in the graph.

To examine the protein level of IL-29 in serum and SF, we measured the concentrations of IL-29 in the serum of RA patients and HC and in SF from patients with RA and OA. Our results showed that serum levels of circulating IL-29 were significantly higher in RA (24.56 ± 15.85 pg/ml) than those in HC (5.62 ± 3.19 pg/ml, *P *<0.0001; Figure 2A). Similar to serum samples, the mean level of IL-29 in SF was increased in RA (16.21 ± 11.12 pg/ml) compared to OA (9.37 ± 4.49 pg/ml) (*P *= 0.0397) (Figure 2B).

To further evaluate the relationship between circulating IL-29 levels and disease activity in RA patients, we next determined the correlations between IL-29 and 28-joint Disease Activity Score (DAS28) as well as laboratory characteristics including CRP, ESR, RF, and anti-CCP. No significant correlation between serum levels of circulating IL-29 and DAS28, CRP, ESR, RF, and anti-CCP was found (data not shown). However, when RA patients were divided into active and inactive groups according to DAS28 scores (that is, the active groups was defined as a DAS28 score ≥3.2 based on the EULAR diagnostic criteria [20]), it was found that active groups conferred moderately higher IL-29 mRNA and protein levels than inactive groups albeit without statistical significance.

### IL-29 expression in synovial tissue

**Figure 3:**
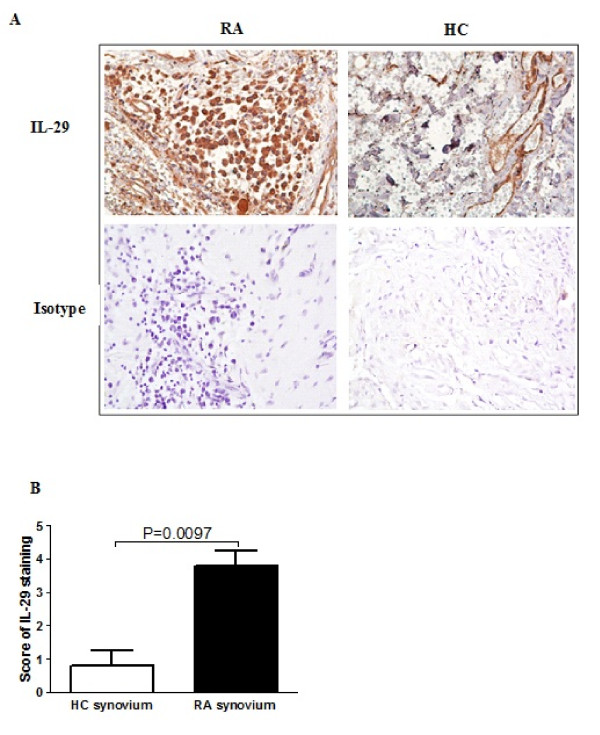
**Increased expression of IL-29 in rheumatoid arthritis (RA) synovium compared to healthy control (HC) synovium**. RA (n = 6) and HC (n = 5) synovium were stained with rabbit anti-human IL-29 **(A)**, and immunostaining was scored on a 0 to 5 scale **(B)**. Values in (B) are the mean ± SD. Actual *P *values are shown in the graph.

Next we sought to examine the localization of IL-29 expression in RA synovial tissues and performed immunohistochemical analysis on six RA and five normal synovial tissue samples. As shown in Figure 3A, IL-29 was mainly expressed in the lining layers of RA synovium. We also semiquatitatively analyzed protein levels of IL-29 expression using a scoring system by immunohistochemistry (Figure 3B), and found that IL-29 expression was significantly increased in RA synovium compared to normal synovium (*P *= 0.0097).

**Figure 4:**
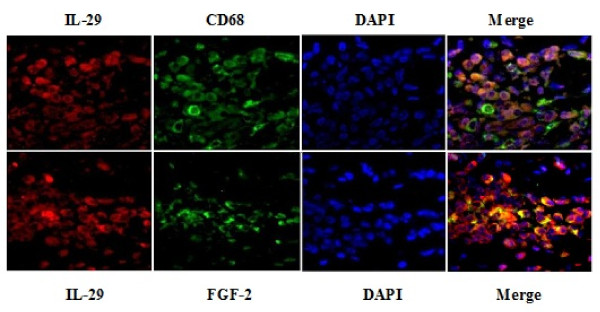
**Cellular distribution of IL-29 in rheumatoid arthritis (RA) synovium**. Double immunofluorescence analysis in RA synovium was performed using specific antibodies against IL-29 and CD68 or FGF-2. Each section was merged with DAPI. The magnification was × 400.

To further determine which cell type expresses IL-29, double immunofluorescence staining was performed in RA synovium using specific antibodies against CD68 and FGF-2 serving as markers of macrophages and fibroblasts, respectively (Figure 4). In the lining layers of the same section used in the above experiments, IL-29-positive cells were stained in red whereas CD68- or FGF-2-positive cells were stained in green. In the merged image, IL-29 positive cells were co-stained with anti-CD68 or anti-FGF-2 as shown in yellow. We found that both macrophages and synovial fibroblasts expressed IL-29, which suggest that these cells might be the important cellular sources of IL-29 in RA synovium.

### Recombinant IL-29-induced cytokine production in MH7A cells

**Figure 5:**
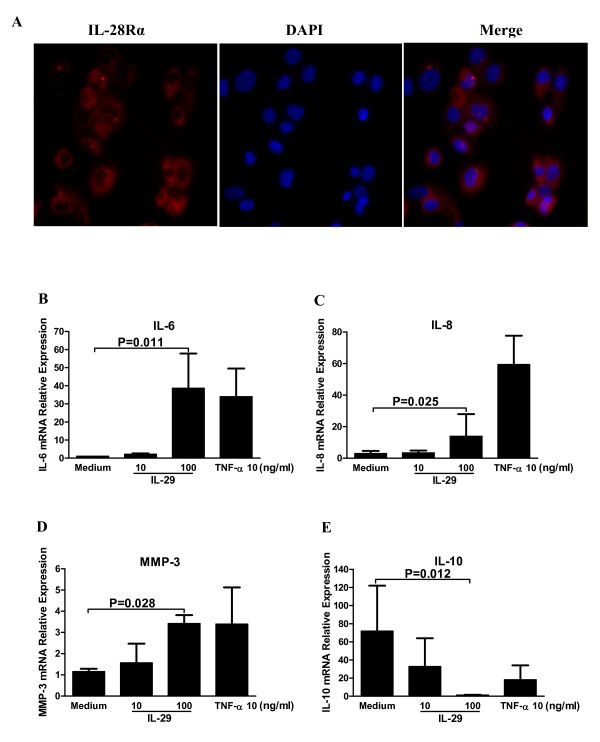
**IL-29 induced cytokine expression by rheumatoid arthritis (RA) synovial fibroblasts**. **(A) **Immunofluoresence staining for IL-28Rα in MH7A cells. IL-28Rα-positive cells were stained red. The magnification was × 400. **(B-E) **Induction of IL-6, -8, -10 and MMP-3 in MH7A cells by IL-29. Levels of mRNA for IL-6 (B), IL-8 (C), MMP-3 (D) and IL-10 (E) in MH7A were determined by real-time PCR after 24 h incubation with IL-29 or TNF-α (a positive control). Because the effects of IL-29 at 1 ng/ml or IL-29 incubation for 48 h on the expression of the above cytokines in MH7A cells are similar to the medium control group or 24 h incubation, the data from IL-29 at 1 ng/ml or IL-29 incubation for 48 h in MH7A are not shown in the figure. IL-29 could not induce the expression of IL-17 in MH7A cells and data are not shown in the figure. Relative gene expression was determined by the 2^-ΔΔct ^method. Values shown are the mean ± SD for five separate experiments performed in triplicate. Actual *P *values are shown in the graph.

IL-28Rα (also known as IFNLR1) is the specific receptor of IL-29. Thus, we first examined the expression of IL-28Ra in MH7A cells. As shown in Figure 5A, IL-28Ra was expressed in MH7A cells, implying that MH7A cells may respond to IL-29 stimulation.

To investigate the potential function of IL-29 in RA, we examined the cytokine profile produced by RA fibroblast cell line-MH7A cells upon IL-29 stimulation. MH7A cells were either untreated or treated with rhIL-29 at concentrations from 1, 10 and 100 ng/ml for 24 h and 48 h, respectively, and TNF-α (10 ng/ml) was used as a positive control. Then, cells were harvested for RT-PCR analysis of IL-6, -8, -10, -17 and MMP-3 expression. As shown in Figure 5, the expression levels of IL-6, -8 and MMP-3 were significantly upregulated in MH7A cells after stimulation with 100 ng/ml rhIL-29 but IL-10 expression was downregulated. Interestingly, IL-17 expression was not changed after 24 h incubation with rhIL-29 at a dose range of 1 to 100 ng/ml in culture (data not shown). Notably, rhIL-29 treatment upregulated the levels of IL-6, IL-8, IL-10 and MMP-3 expression in a dose-dependant fashion, but not in a time-dependant fashion after 24 or 48 h incubation (48 h data not shown).

## Discussion

IL-29 is a new member of type III IFN family of cytokines that has been shown to be involved in immune responses including inhibition of viral infection and proliferation of tumor cells [12,23]. However, the role of IL-29 in the pathogenesis of RA remains unknown. In this study, we characterized, for the first time, IL-29 expression in blood and SF of RA patients. We found that mRNA levels of IL-29 and its specific receptor IL-28Rα in PBMC were significantly higher in RA than those in HC. Levels of IL-29 were greatly elevated in RA serum compared with HC. Moreover, IL-29 levels were significantly higher in RA SF than that in OA SF. Together, these data suggest that IL-29 expression is dysregulated with potentially enhanced biological function in patients with RA. Therefore, we further examined the correlation between blood IL-29 levels and disease activity in RA, together with several laboratory values. However, we did not find any significant difference of IL-29 level in blood between RA patients with active and inactive disease, and serum IL-29 protein levels were also not correlated well with DAS28 or CRP, ESR, anti-CCP and RF levels. Given IL-29 has been shown to induce apoptosis and suppress the cell proliferation of human CD4+ T cells [24,25], it is possible that IL-29 may interact with other pathways involved in RA pathogenesis in addition to inflammation. Alternatively, the lack of a significant association may be attributed to the modest sample size, the restriction in the range of the measure of disease activity and the high standard deviations of CRP and ESR value in our patients. Hence, further prospective and multicenter studies with a larger sample size are needed to determine whether IL-29 can serve as a biomarker for disease activity for RA patients.

A characteristic feature of RA is the hyperplasia of the synovial tissue, resulting from the infiltration of various types of immune cells secreting numerous cytokines, chemokines, and matrix metalloproteinases (MMPs) that promote inflammation and joint destruction through autocrine and paracrine mechanisms [26]. In particular, synovial fibroblasts and macrophages have been identified as the major cell population in the synovial tissue for overproduction of both proinflammatory cytokines and MMPs. Therefore, we further examined whether local IL-29 expression in synovial tissue is dysregulated and how IL-29 modulates synovial inflammation in RA patients. Our results showed for the first time that the number of IL-29-positive cells in RA synovium lining was substantially higher than that in HC. Notably, we identified both synovial macrophages and fibroblasts as the major cellular source for IL-29 expression in RA synovial tissue.

Furthermore, we investigated the function of IL-29 in RA synovial fibroblasts *in vitro *and found that rIL-29 could activate human synovial fibroblasts to produce cytokines IL-6, IL-8 and MMP-3, which may promote inflammation and joint destruction in RA. However, rIL-29 failed to induce IL-17 expression, suggesting little effect of IL-29 on inducing IL-17 production. Several studies have demonstrated the ability of IL-29 to regulate cytokine production in both peripheral blood mononuclear cells and dendritic cells upon viral infections or activation via toll-like receptor-mediated signaling [14,15,25]. Our current findings have suggested that IL-29 is able to activate RA synovial fibroblast cells to produce proinflammatory cytokines. However, further studies are needed to determine whether IL-29 can stimulate cytokine production in primary synovial fibroblasts from RA patients.

Based on current studies, it is interesting to compare the role of different types of IFNs in the pathogenesis of RA. Lines of evidence have indicated that type I IFNs play an important role in the pathogenesis of RA. Type I IFN-related genes were significantly increased in PBMC of RA patients [27], whereas IFN-α and IFN-β were upregulated in the synovium of RA [28,29]. However, IFN-γ was lacking, or at low levels in the synovium, and rarely detectable in the SF of patients with RA [30,31]. Therefore, compared with above research findings, we found that there were some similarities with respect of the expression and mechanism between IL-29 and type I IFNs in the pathogenesis of RA. As a potential therapeutic agent in the treatment of viral infections and cancers, IL-29 has attracted new interest for research, because the tissue-restricted expression of IL-29 and its receptor make IL-29 therapy have fewer side effects than type I IFNs therapy that is accompanied by numerous side effects [7].

## Conclusions

In summary, our data have presented new evidence that IL-29 may contribute to synovial inflammation during RA pathogenesis. Further studies on the induction of IL-29 production and its underlying molecular mechanisms will provide a fuller understanding of a pathological role of IL-29 in RA development.

## Abbreviations

BSA: bovine serum albumin; CRP: C-reactive protein; DAPI: 40, 6-diamidine-20-phenylindole dihydrochloride; DAS28: 28-joint Disease Activity Score; DMEM: Dulbecco's modified Eagle's medium; ELISA: enzyme-linked immunoassay; ESR: erythrocyte sedimentation rate; FCS: fetal calf serum; HC: healthy controls; IFNs: interferons; IL-29: interleukin-29; mAB: monoclonal antibody; MMP-3: matrix metalloproteinase-3; OA: osteoarthritis; pAB: polyclonal antibody; PBMC: peripheral blood mononuclear cells; PBS: phosphate-buffered saline; RA: rheumatoid arthritis; RF: rheumatoid factor; rhIL-29: recombinant human IL-29; RT-PCR: real-time polymerase chain reaction; SF: synovial fluid; TNF-α: tumor necrosis factor-alpha.

## Competing interests

The authors declare that they have no competing interests.

## Authors' contributions

FW, WT and MZ contributed to the study design, data analysis and manuscript preparation. LX, XF and DG performed experiments. All authors read and approved the final manuscript.
